# Unraveling Differences in Molecular Mechanisms and Immunological Contrasts between Squamous Cell Carcinoma and Adenocarcinoma of the Cervix

**DOI:** 10.3390/ijms25116205

**Published:** 2024-06-05

**Authors:** Morteza Salarzaei, Ralf L. O. van de Laar, Patricia C. Ewing-Graham, Shiva Najjary, Edith van Esch, Heleen J. van Beekhuizen, Dana A. M. Mustafa

**Affiliations:** 1 Department of Gynaecologic Oncology, Erasmus MC Cancer Institute, Erasmus University Medical Center, 3015 GD Rotterdam, The Netherlandsh.vanbeekhuizen@erasmusmc.nl (H.J.v.B.); 2 Department of Pathology and Clinical Bioinformatics, Erasmus University Medical Center, Dr. Molewaterplein 40, 3015 GD Rotterdam, The Netherlands; 3 Department of Pathology and Clinical Bioinformatics, The Tumor Immuno-Pathology Laboratory, Erasmus University Medical Center, Dr. Molewaterplein 40, 3015 GD Rotterdam, The Netherlands; s.najjary@erasmusmc.nl; 4 Department of Gynecology and Obstetrics, Catharina Ziekenhuis Eindhoven, Michelangelolaan 2, 5623 EJ Eindhoven, The Netherlands; edith.v.esch@catharinaziekenhuis.nl

**Keywords:** cervical cancer, targeted gene expression profiling, squamous cell carcinoma, adenocarcinoma, immune cells

## Abstract

This study aims to refine our understanding of the inherent heterogeneity in cervical cancer by exploring differential gene expression profiles, immune cell infiltration dynamics, and implicated signaling pathways in the two predominant histological types of cervix carcinoma, Squamous Cell Carcinoma (SCC) and Adenocarcinoma (ADC). Targeted gene expression data that were previously generated from samples of primary cervical cancer were re-analyzed. The samples were grouped based on their histopathology, comparing SCC to ADC. Each tumor in the study was confirmed to be high risk human papilloma virus (hrHPV) positive. A total of 21 cervical cancer samples were included, with 11 cases of SCC and 10 of ADC. Data analysis revealed a total of 26 differentially expressed genes, with 19 genes being overexpressed in SCC compared to ADC (Benjamini–Hochberg (BH)-adjusted *p*-value < 0.05). Importantly, the immune checkpoint markers CD274 and CTLA4 demonstrated significantly higher expression in SCC compared to ADC. In addition, SCC showed a higher infiltration of immune cells, including B and T cells, and cytotoxic cells. Higher activation of a variety of pathways was found in SCC samples including cytotoxicity, interferon signaling, metabolic stress, lymphoid compartment, hypoxia, PI3k-AKT, hedgehog signaling and Notch signaling pathways. Our findings show distinctive gene expression patterns, signaling pathway activations, and trends in immune cell infiltration between SCC and ADC in cervical cancer. This study underscores the heterogeneity within primary cervical cancer, emphasizing the potential benefits of subdividing these tumours based on histological and molecular differences.

## 1. Introduction

Cervical carcinoma (CC) continues to be a public health concern, despite the adoption of early detection protocols. Cervical cancer rates have declined in developed countries due to effective screening programs. However, in developing nations, there is a concerning increase in prevalence, attributed in part to inadequate screening and follow-up. Globally, there are 341,000 deaths per year from this malignancy, underlining its significant impact on women’s health [1,2,3,4].

Persistent infection with high-risk human papillomavirus (HPV) is the predominant cause of CC, accounting for at least 98% of cases [4,5]. HPV impedes the inflammatory response in the epithelium, leading to diminished cytokine and chemokine production. This results in reduced attraction of dendritic cells, allowing the virus to bypass the immune system and remain in the body [6]. It is not yet fully understood why HPV persists in one individual while being cleared in another.

Cervical cancers predominantly fall into two histological subtypes, squamous cell carcinoma (SCC) and adenocarcinoma (ADC) [7]. These arise from different cell types in the cervix, including squamous epithelial cells found in the ectocervix and mucin-containing cylindrical cells found in the endocervix. Although these cancers emerge from distinct cell types within the same organ, they are often treated similarly. This variation in cell of origin may contribute to the differences observed in tumor microenvironment (TME) and immunological pathways [8].

The TME, a complex and dynamic network of neoplastic, immune, stromal and endothelial cells, along with extracellular matrix (ECM) components, is increasingly recognized as influential in the course of cancer progression and in response to therapy [9]. The unique molecular and genetic characteristics of SCC and ADC lead to distinct TMEs, with potential implications for the immune response to these cancers [10]. Understanding the specific mechanisms of immune evasion employed by SCC and ADC is crucial, given the growing interest in immunotherapy as a promising avenue for cervical cancer treatment [10].

There are very limited studies focusing on the differences between cervical SCC and ADC with respect to molecular and immunological aspects. These studies highlighted differences in the expression of PD-1/PDL-1, as well as the activation of IL-17, JAK/STAT, and Ras signaling pathways between cervical SCC and ADC [10,11]. While existing research has provided valuable insights into the diversity and complexity of cancers, there remains a need for more in-depth studies. This is particularly true for advancing personalized treatment strategies for more targeted therapies in SCC and ADC. The aim of this study was to evaluate and compare the differential gene expression profiles, immune cell infiltration dynamics, and implicated signaling pathways between cervical SCC and ADC.

## 2. Results

A total of 21 primary cervical cancer stage Ib1-2 (FIGO 2009) samples were included for data analysis. The samples were divided into the following two distinct groups based on the histopathology status: Squamous Cell Carcinoma (SCC, *n* = 11) and Adenocarcinoma (ADC, *n* = 10). The clinical values of the two groups, including age, HPV status and other details, were similar and did not show any significant variation (Table 1). The mean invasion depth of SCC was 8.22 ± 4.1 mm and of ADC was 7.07 ± 3.3 mm, (*p*-value = 0.49). 

### 2.1. Differentially Expressed Genes in SCC and ADC

**Figure 1:**
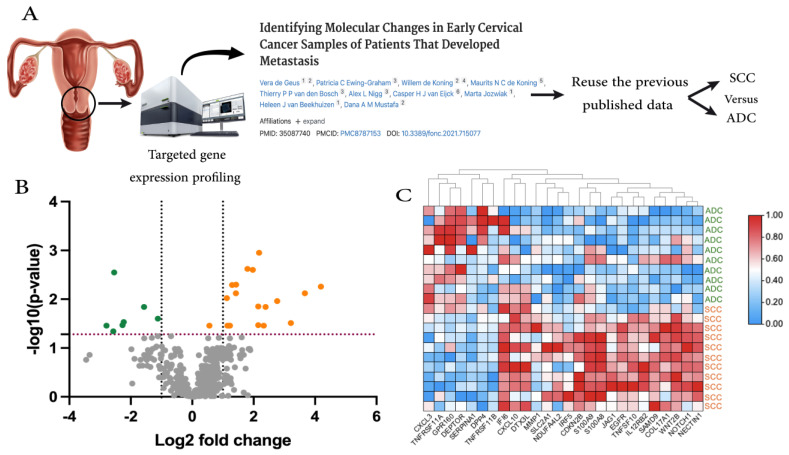
Overview of the study and the findings. (**A**) Schematic representation of the study design [1]. (**B**) Volcano plot of the differentially expressed genes between Squamous Cell Carcinoma (SCC) and Adeno Cell Carcinoma (ADC). The dotted red line presents the BH-*p*-value < 0.05. The horizontal dotted lines present the Fold of Change (FOC) > 1. Every dot presents a measured gene, while the orange dots are DEG higher in SCC, green dots are DEG lower in SCC (BH, *p*-value < 0.05) and gray dots are not significantly different between the two groups. (**C**) Heat-map of the DEG genes that were filtered additionally by FOC > 1.

Analyzing the gene expression data revealed a distinct difference in pattern between SCC and ADC. A total of 19 genes were significantly overexpressed and 7 were under-expressed genes in SCC (BH, *p*-value < 0.05) (Figure 1B and Table 2). Around 20 of the differentially expressed (DE) genes showed a high Fold-of Change (FOC) value (log2-fold > 2 or <−2) (Figure 1C).

### 2.2. The Immune Landscape of SCC and ADC

**Figure 2:**
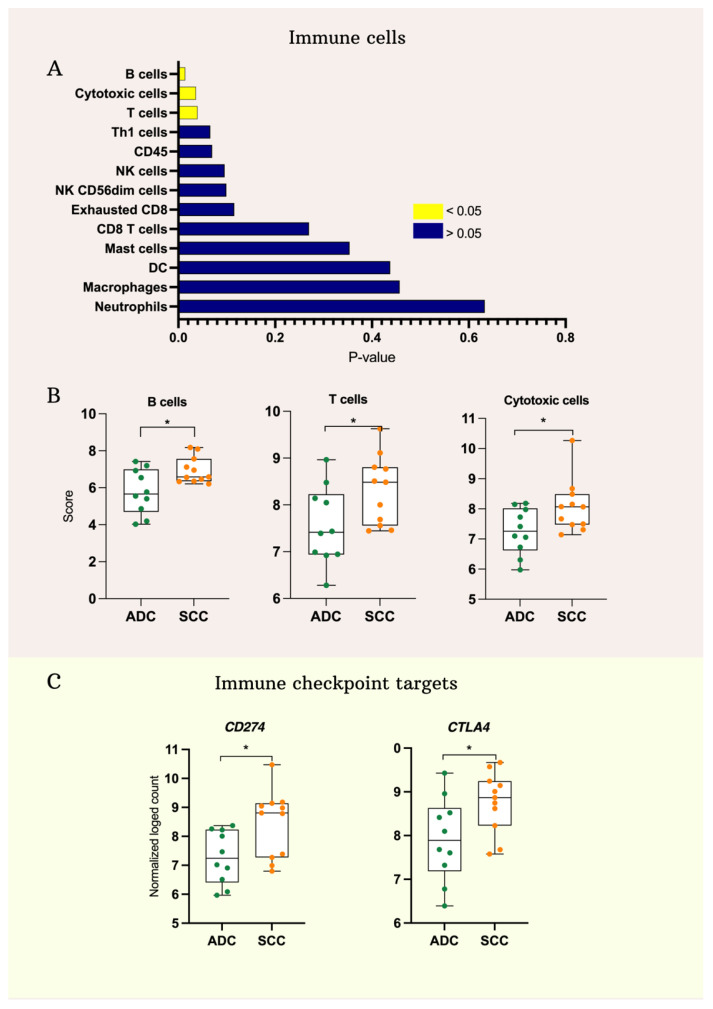
The immune landscape of cervical Squamous Cell Carcinoma (SCC) and Adeno Cell Carcinoma (ADC). (**A**) Bar plot of the *p*-values resulted from the comparison of immune cell infiltration scores. Yellow bars are for the significant scores and the blue bars are for the not significant scores. (**B**) Box plots for significantly abundant immune cells in SCC. (**C**) Box plots of the differentially expressed immune checkpoint targets. (* *p*-value < 0.05).

Pairwise similarities were used to identify immune cells in tissue samples as described previously [1]. A higher number of B cells, T cells and cytotoxic cells was found in SCC compared to ADC (Figure 2A,B). However, all the other identified cells such as NK cells, mast cells, dendritic cells, macrophages and neutrophils showed similar numbers in the two groups. Importantly, the immune checkpoint markers *CD274* and *CTLA4* were found to be significantly more highly expressed in SCC (Figure 2C).

### 2.3. Higher Activation of the Immune-Related Pathways in SCC Samples Compared to ADC

**Figure 3:**
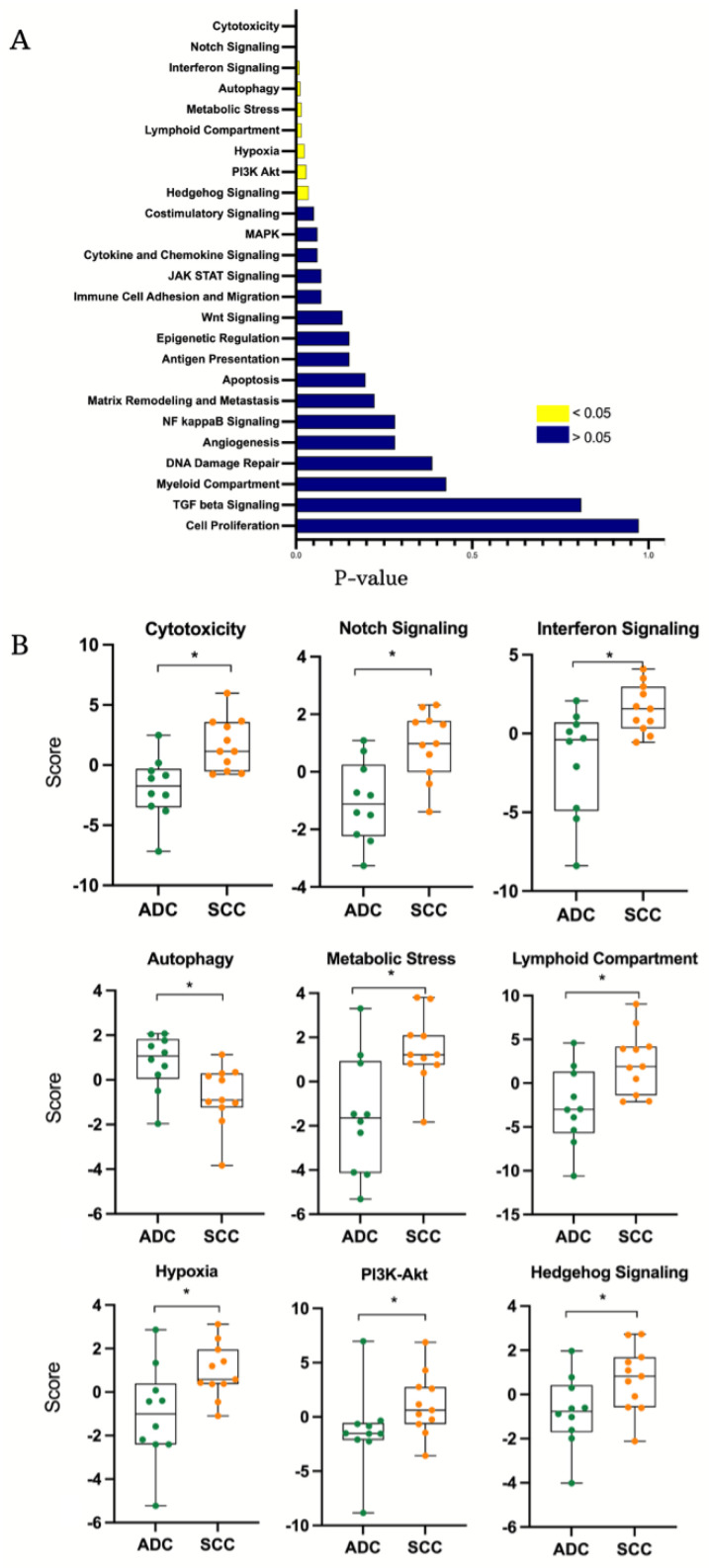
Pathway scores for Squamous Cell Carcinoma (SCC) and Adeno Cell Carcinoma (ADC). Bar plot of the *p*-values resulted from the (**A**) bar plot of the *p*-values resulted from the comparison of pathway scores. Yellow bars are for the significant scores and the blue bars are for the not significant scores. (**B**) Box plots for the following significant pathways: cytotoxicity, notch signaling, interferon signaling, autophagy, metabolic stress, lymphoid compartment, hypoxia, PI3K-Akt, and hedgehog signaling. (* *p*-value < 0.05).

**Figure 4:**
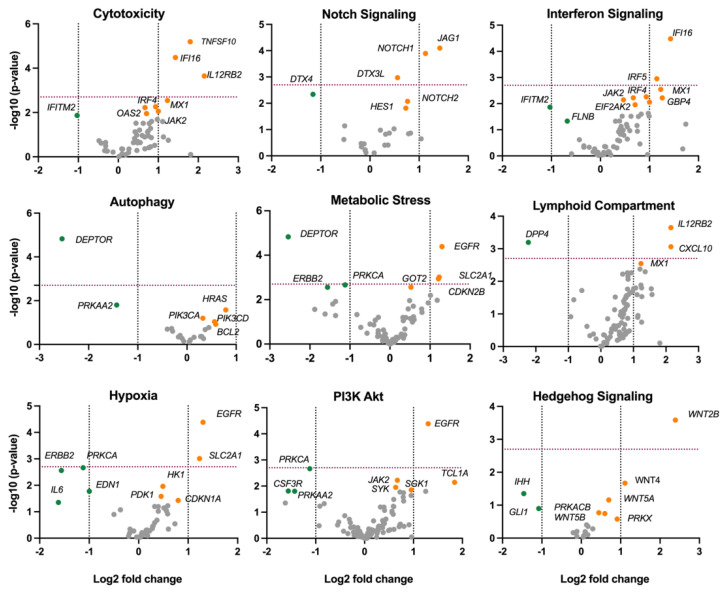
Comparing the volcano plots of GSA for cytotoxicity, notch signaling, interferon signaling, autophagy, metabolic stress, lymphoid compartment, hypoxia signaling, PI3K-Akt, and hedgehog signaling pathways between SCC (orange dots) and ADC (green dots). The dashed red line shows significance level of *p* value.

Pathway analysis of the profiled genes revealed significant activation of several signaling pathways in SCC compared to ADC. Notably, the cytotoxicity pathway was more activated in SCC (BH, *p*-value < 0.002), as were notch signaling (BH, *p*-value < 0.003), interferon signaling (BH, *p*-value < 0.001), metabolic stress (BH, *p*-value < 0.015), lymphoid compartment (BH, *p*-value < 0.016), hypoxia (BH, *p*-value < 0.024), PI3k-AKT (BH, *p*-value < 0.029) and hedgehog signaling (BH, *p*-value = 0.03). On the other hand, only the autophagy pathway scored more highly in ADC compared to SCC (BH, *p*-value = 0.012) (Figure 3B). Additional Gene Set Enrichment Analysis (GSEA) showed genes involved in the pathway analysis and provided key insights into the unique molecular dynamics underlying these two cancer subtypes (Figure 4).

## 3. Discussion

While the histopathological typing of cervical cancer is routinely documented in pathology reports, its translation into clinical or therapeutic strategies has been lacking. In our study, we identified 26 DE genes between SCC and ADC, 19 of which were found to be highly expressed in SCC. Notably, SCC exhibited heightened immune stimulation, evident in increased immune cell infiltration and elevated scores in immune-related pathways compared to ADC. Crucially, immune checkpoint targets *CD274* and *CTLA4* demonstrated elevated expression in SCC. It is important to note that our study selected a highly homogeneous group of primary cervical cancer samples. Our findings suggest the possible subdivision of primary cervical cancer into, at least, two distinct groups discernable at both molecular and immunological levels. Our findings suggest that SCC and ADC can be subdivided not only on grounds of morphology but also at the molecular and immunological level. Furthermore, our data support the consideration of specific anti-CD274 and anti-CTLA4 drugs in the treatment regimen for SCC, offering a tailored therapeutic approach.

The results of our study are similar to previous findings. The study by Campos Parra et al. compared the molecular features of cervical SCC and ADC by analyzing data from The Cancer Genome Atlas (TCGA) and a Mexican-Mestizo dataset. They identified 70 consistently DEGs across both datasets, associated with pathways such as IL-17 and JAK/STAT [10]. Our DEGs did not overlap with the DEGs of Campos Parra et al.’s study because the measured genes were different. However, both studies suggest that there is an intricate web of signaling pathways influencing the progression and characteristics of cervical cancer, with certain pathways potentially being more specific to SCC or ADC. Notably, both studies identified signaling pathways (such as Notch1 in the current study and JAK/STAT from the previous study) that play a pivotal role in cell growth, differentiation, and immune responses in cancers [10]. A study by Wild et al. in which they compared the level of tumor-infiltrating lymphocytes (TILs) between cervical SCC and ADC found that SCC displayed an increased level of TILs [12]. Additionally, a study by Karpathiou et al. that focused on investigating immune checkpoints revealed that SCC exhibited higher expression of both CTLA-4 and PD-L1 [13].

There are also studies comparing the immune-related genes in lung/esophagus SCC and ADC, which found several immune-associated prognostic DEGs. These results indicate the need for tailored therapeutic strategies based on subtype-specific molecular signatures [14,15].

Lin et al. in their study comparing genetic and immunologic aspects of SCC and ADC in different organs reported that gene expression profiles that are determined based on histology are generally the same across the organs. In keeping with our study, they also found DEGs distinguishing SCC from ADC, emphasizing the importance of understanding the molecular differences between these subtypes [16].

Another important factor differentiating these two subtypes can be the HPV type and status. Research on vulvovaginal squamous cell carcinoma indicates that HPV-associated tumors frequently harbor PIK3CA mutations, whereas HPV-independent cancers often exhibit alterations in TERT, TP53, and CDKN2A, emphasizing distinct molecular pathways influenced by HPV types. In particular, HPV 18, which is more frequently associated with adenocarcinoma, may induce distinct molecular alterations compared to HPV 16, commonly linked with squamous cell carcinoma. Studies have demonstrated that HPV 18-related adenocarcinomas exhibit fewer pre-cancerous lesions, suggesting a more direct progression to invasive cancer, which could be attributed to unique gene expression profiles influenced by the viral oncogenes. These findings indicate that the carcinogenic mechanisms of HPV 18 could differ significantly from those of HPV 16, potentially impacting the gene expression landscape and, subsequently, the therapeutic responses [17,18]. This is in line with the study by Preti et al., which reported that different HPV types target different cells and exhibit distinct carcinogenic patterns, influencing the progression of vaginal carcinoma. Their research indicates that HPV-associated carcinogenic mechanisms vary by HPV type, which could impact gene expression profiles and therapeutic responses. These variations highlight the importance of considering HPV genotype when developing targeted treatment strategies [19].

Furthermore, the pronounced immune cell infiltration observed in SCC as compared to ADC may be influenced by the differential immune modulation by HPV types commonly associated with these subtypes. This aligns with findings from Kobayashi et al., which highlight the crucial role of HPV-specific immune responses in shaping the tumor microenvironment. Their review suggests that HPV 16, commonly associated with SCC, may induce a stronger Th1-mediated cellular immune response, which could explain the enhanced immune cell infiltration in SCC observed in our findings [20]

Despite the significance of our findings, it is important to acknowledge that the sample size is limited. Validation using a larger independent cohort of samples would be an important next step. Our study’s primary strength lies in its detailed exploration of molecular and immunological differences between cervical squamous cell carcinoma (SCC) and adenocarcinoma (ADC), identifying 26 differentially expressed genes. This discovery is pivotal for understanding the complex biology of cervical cancer, setting a foundation for targeted therapeutic strategies based on molecular profiles.

The implications of our findings are significant for clinical practice, suggesting that molecular profiling could lead to more personalized treatment approaches for cervical cancer. Specifically, our data advocate for the consideration of therapies targeting CD274 and CTLA4 in SCC patients, marking a step towards precision oncology that could substantially improve patient outcomes.

## 4. Materials and Methods

### 4.1. Samples

This study builds upon our previous work that included 34 samples of primary cervical cancer patients and 12 samples of a normal cervix [1]. Targeted gene expression profiles of cancer- and immune-related genes were generated from all the samples.

For the current study, we selected the most clinically homogeneous group of samples consisting of 21 primary cervical cancer cases that did not develop distant recurrence. All the samples were stage Ib1-2 (FIGO 2009) at primary diagnosis, and all were hrHPV positive. To address the aim of this study, samples were grouped based on histopathology diagnosis. Therefore, the gene expression profile of 11 SCC samples was compared to that of 10 ADC samples (Figure 1A).

### 4.2. Statistical Analysis

The previously generated targeted expression profiles using the IO 360 panel of nanoString were reanalyzed [1]. Expression data were re-uploaded into the nSolver software (version 4.0), and the advanced analysis module (version 2.0, NanoString, Seattle, WA, USA) was used for analysis. The raw gene counts were normalized using the most stable housekeeping genes present in the panel, chosen via the geNorm algorithm [21]. The background threshold was set by calculating twice the mean count of the eight negative controls. Genes with counts surpassing this threshold were deemed as detected. Differentially expressed (DE) genes were calculated using a mixture of binomial models, log linear model, or a simplified negative binomial model. The results were considered significant after applying Benjamini-Hochberg correction (BH). BH-*p*-value < 0.05 was considered significant. Variations in pathway and cell type scores were examined using the Mann–Whitney U test. Graphical representations including heatmaps and volcano plots were generated using the TBtools software (version 1.055) [22]. The (R software) (version 4.4.0) was employed for data visualization and correlation analyses.

## 5. Conclusions

Primary cervical cancer can be divided into squamous cell carcinoma and adenocarcinoma at both the histopathological and molecular level. Cervical SCC exhibits significantly higher immunogenicity compared to ADC. In addition, SCC expresses significantly higher levels of s*CD274* and *CTLA4*, which highlights the possibility of using targeted therapy to treat SCC of the cervix.

## Figures and Tables

**Table 1:** Clinical characteristics of included patients.

Characteristics		Total (*n* = 21)	SCC (*n* = 11)	ADC (*n* = 10)	*p*-Value
Age (mean)			42.73 ± 8.5	41.60 ± 7.7	0.75
Type of radical hysterectomy	Open	12 (57%)	7 (63%)	5 (50%)	0.69
	Robot	9 (43%)	4 (37%)	5 (50%)	
Type of HPV	16	14 (66%)	10 (91%)	4 (40%)	0.02
	18	5 (24%)	0	5 (50%)	
	16 and 18	1 (5%)	0	1 (10%)	
	52	1 (5%)	1 (9%)	0	
Invasion depth			8.22 ± 4.1	7.07 ± 3.3	0.49
LVSI	Yes	9 (43%)	6 (54%)	3 (30%)	0.38
	No	12 (57%)	5 (46%)	7 (70%)	
Recurrent disease after primary treatment	No	11 (52%)	5 (45%)	6 (60%)	0.67
	Local	10 (48%)	6 (55%)	4 (40%)	

**Table 2:** A summary of the DEGs in SCC and ADC.

Gene Over-Expression	BH *p*-Value	*p*-Value	Linear Fold Change
Upregulated SCC compared to ADC			
*NECTIN1*	0.001	0.0000015	4.54
*TNFSF10*	0.002	0.00000643	3.49
*SAMD9*	0.002	0.0000101	3.92
*IFI16*	0.004	0.0000331	2.7
*EGFR*	0.005	0.0000411	2.45
*S100A8*	0.005	0.0000517	18.3
*JAG1*	0.007	0.0000799	2.67
*S100A9*	0.007	0.0000908	12.7
*NOTCH1*	0.009	0.000090	2.19
*NDUFA4l2*	0.01	0.000159	6.82
*IL12RB2*	0.014	0.000224	4.43
*WNT2B*	0.0146	0.00026	5.25
*MMP1*	0.03	0.000699	9.25
*CXCL10*	0.03	0.000866	4.45
*SLC2AL*	0.03	0.000977	2.35
*COL17A1*	0.03	0.00104	5.04
*DTX3L*	0.03	0.00106	1.47
*IRF5*	0.03	0.00112	2.22
*CDKN2B*	0.034	0.00115	2.31
Downregulated ADC compared to SCC			
*DEBTOR*	0.002	0.0000149	5.81
*GPR160*	0.01	0.000272	2.96
*TNFRSF11A*	0.025	0.000501	2.16
*DPP4*	0.029	0.000636	4.69
*CXCL3*	0.03	0.000932	6.89
*SERPINA1*	0.033	0.000806	4.83
*TNFRSF11B*	0.045	0.00158	5.91

## Data Availability

The study’s data have been made available in the Gene Expression Omnibus (GEO) database, under the accession code GSE192897.
